# Surface-Selective Molecular Binding and Replacement Selectivity in Plasmonic Nanocavities

**DOI:** 10.1021/acs.jpclett.6c00963

**Published:** 2026-05-07

**Authors:** Eric S. A. Goerlitzer, Zijia Wu, Aidan Brzakalik, Shu Hu, Bart de Nijs, Jeremy J. Baumberg

**Affiliations:** † Nanophotonics Centre, Cavendish Laboratory, Department of Physics, 2152 University of Cambridge, Cambridge CB3 0US, United Kingdom; ‡ Physics for Sustainable Chemistry Group, Cavendish Laboratory, Department of Physics, University of Cambridge, Cambridge CB3 0US, United Kingdom

## Abstract

Molecular self-assembled monolayers (SAMs) have a strong role in nanoscience and nanotechnology, being exploited for sensing, molecular electronics, catalysis, spin transport, and more. Typically, thiol binding to coinage metals produces well-ordered, robust molecular layers. While their replacement by substituting thiols has been studied on planar surfaces, little is yet known when the SAMs are confined at the nanoscale. Here, using strong plasmonic confinement, we optically track how thiol SAMs are replaced in nanocavities and show that an unexpected mechanism is introduced when nanoparticles are placed on top of the SAM. Using a range of model molecules demonstrates that replacement thiols preferentially attach to the nanoparticle and are rotated into the nanogap. These dynamics can be selectively prevented using dithiols that fix facet metal atoms in place. This mechanism offers a promising route for spatially selective chemical control, enabling asymmetric molecular architectures and postdeposition functionalization of plasmonic nanostructures.

## Graphical abstract

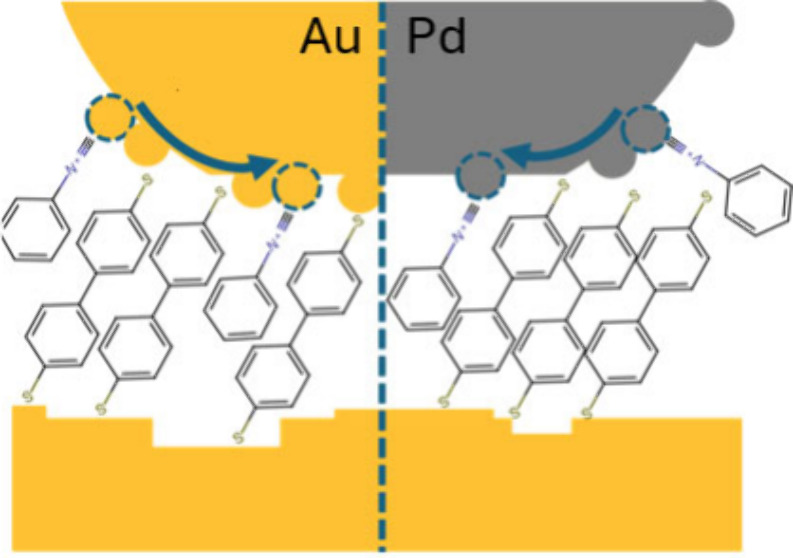

Self-assembled monolayers (SAMs) are molecular assemblies that spontaneously organize on metal surfaces, providing a robust method for tailoring surface properties with molecular-level precision.^1–3^ A notable characteristic of SAMs is their dynamic exchange behavior: upon exposure to a solution containing different anchor molecules, the original monolayer can be progressively displaced, facilitating chemical reprogramming of the interface and the formation of multicomponent monolayers.^4–6^ This exchange process has been extensively utilized in biosensing and molecular electronics, where introducing chemical diversity and tuning interfacial properties are crucial for device functionality.^4,7–12^

Extensive studies have shown that the resulting multicomponent SAM composition is governed by many factors including adsorption kinetics, molecular size, intermolecular interactions (such as van-der-Waals, π–π stacking), compactness, and the relative binding affinities of the competing species.^3,6,13^ Experimental observations, including surface plasmon resonance, scanning tunnelling microscopy, and spectroscopic techniques, have confirmed that exchange on flat gold surfaces is efficient and often leads to either homogeneous or phase-separated mixed SAMs.^14–16^ Despite extensive understanding of SAM behavior on extended planar substrates, much less is known about SAM behavior in nanoscale confined environments, such as nanocavities, where spatial restrictions and limited solvent access are expected to fundamentally alter molecular dynamics. Recent work has suggested that ligand-spaced nanoparticle-on-mirror nanogaps can behave as quasi-two-dimensional nanochannels supporting molecular infiltration and exchange.^17^ However, the microscopic mechanism by which molecules initially access these confined cavities and their binding preference to the gap remains unresolved, representing a more fundamental question. This is especially critical in plasmonic nanogap environments, where molecular composition directly influences optical properties, field enhancement, and chemical reactivity. Such systems form vital components for molecular electronics, photocatalysis, liquid or gas sensing in healthcare, environmental monitoring, and many other devices.

To better investigate molecular exchange in nanoconfined environments, we employ a series of well-defined nanoparticle-on-mirror (NPoM) constructs.^18–21^ In this system, an 80 nm gold faceted-spherical nanoparticle (NP) is positioned above a flat gold substrate, separated by a nanometer gap defined by the SAM coating on the underlying Au substrate (Figure 1a). The nanogap supports intense electromagnetic hotspots giving intense surface-enhanced Raman scattering (SERS) signals from the molecules, while its structural simplicity allows precise control over the molecular environment. The scattering spectra from each NPoM show strong resonances in scattering, whose wavelengths depend on the gap size, refractive index, NP size, and faceting.^22^ Typically, each NPoM shows a slightly different main resonance λ_c_ (from the mode labeled (10)) due to variations in NP morphology; hence, histograms of many (>100) particle spectra are recorded in each case. We use SAMs of biphenylthiol (BPT, **B**), terphenylthiol (TPT, **T**), biphenyldithiol (BPDT, **D**), and phenyl isocyanide (PIC, **P**) as molecular probes (Figure 1b), each offering distinct size and spectroscopic signatures that enable detailed tracking of molecular binding and replacement.

**Figure 1:**
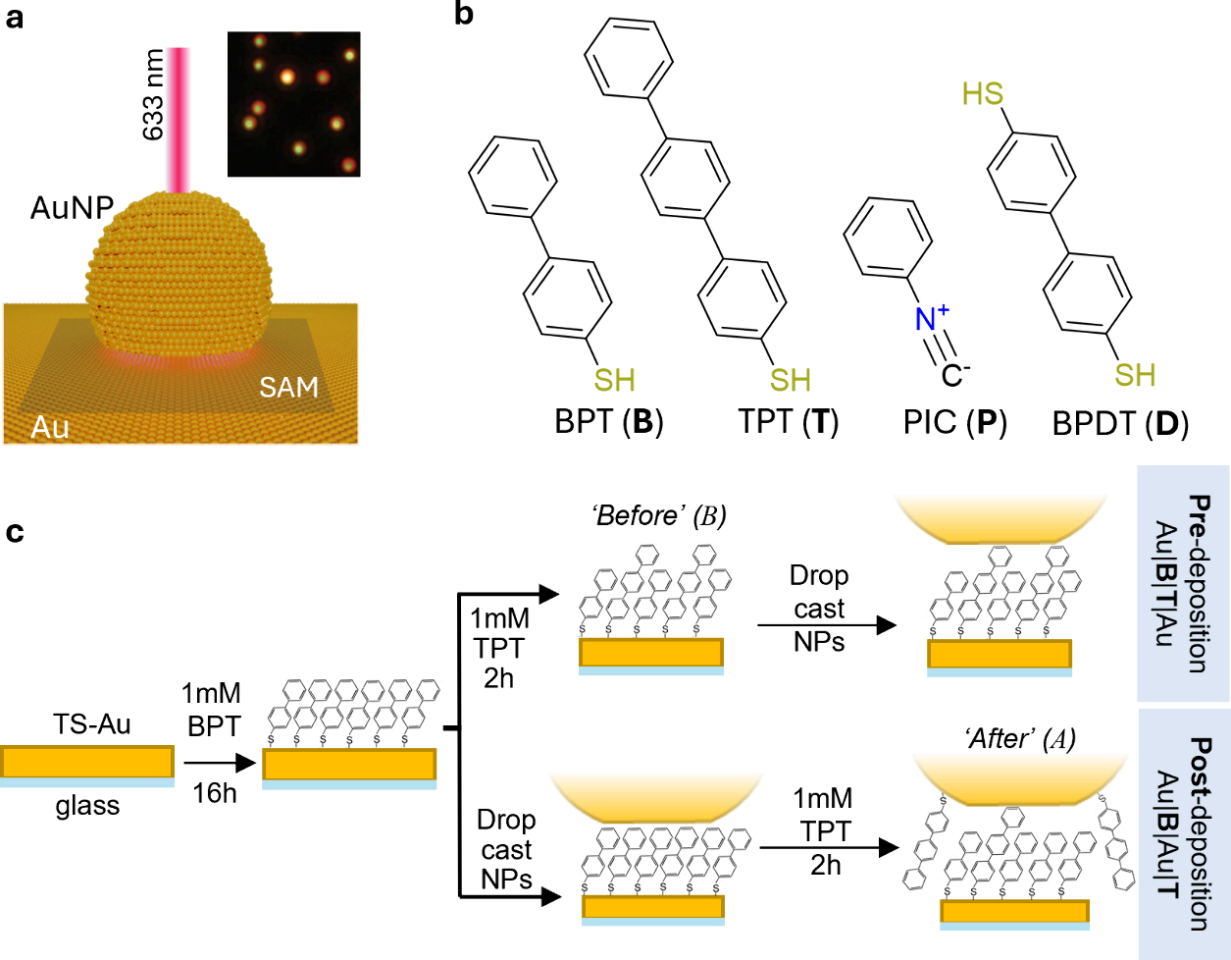
(a) Schematic of NPoM construct. Inset shows the dark field image of BPT SAM on gold with AuNPs on top. (b) Molecules used in SAM formation and replacement; see text. (c) Pre- and postdeposition processes for making multimolecular SAMs inside NPoMs, denoted Au|B|T|Au and Au|B|Au|T, depending on whether replacement is before (*B*) or after (*A*) the AuNP is placed on top.

By combining SERS and dark-field (DF) scattering spectroscopy, we systematically compare the behavior of these molecules both inside nanocavities and on bulk planar gold. While SAMs on flat surfaces undergo rapid and efficient exchange, molecules within the nanogap are found to exhibit exceptional stability, resisting replacement even when exposed to high concentrations of competing species. Instead, new molecules are found to bind selectively to the nanoparticle surface, leaving the gap-defining SAM mostly intact. This spatially selective molecular behavior, substituting at the bare surface but resistant within the gap, highlights unique confinement effects of nanogap geometries in molecular exchange and opens pathways for postfabrication functionalization and asymmetric molecular patterning in widespread nanodevices.

## Binding and Replacement within the Nanocavity vs Planar Au

We employ a stepwise assembly strategy in which gold substrates are first functionalized with a primary SAM (e.g., M_1_ = BPT). This is followed (Figure 1c; SI Methods) by solution immersion in the second thiolated species M_2_ before deposition of gold nanoparticles which takes place either before (pre-deposition, denoted Au|M_1_|M_2_|Au, see Figure 1) or after NP deposition (post-deposition, denoted Au|M_1_|Au|M_2_). This compares how competition between the two molecules M_1,2_ depends on their environment. In all cases, we find that replacement happens rapidly (within a few minutes) and saturates with no further changes over several hours.

We thus create six types of NPoM construct (Figure 2a) and systematically compare how their assembly order affects nanogap composition and optical response (Figure 2b–d). SERS spectra without molecular replacement (Figure 2 rows i and iv) show the vibrational fingerprints of the two SAMs when they bind on the lower Au substrate. In particular, distinct peaks arise from a BPT mode at 656 cm^–1^ and a TPT mode at 645 cm^–1^ (Figure 2c,d), among others. Upon replacement, fitting these peak areas allows extraction of the fraction of each molecule present (compared to their pure SAM alone) within the optical hotspot of ∼5 nm lateral width under the facet center.^22^ We note minimal changes in vibrational peak line width, suggesting that SAM intermolecular order is little affected. We also note that SERS from molecules outside the nanogap contribute <0.1% of signals due to the strong optical field confinement.^22^

**Figure 2:**
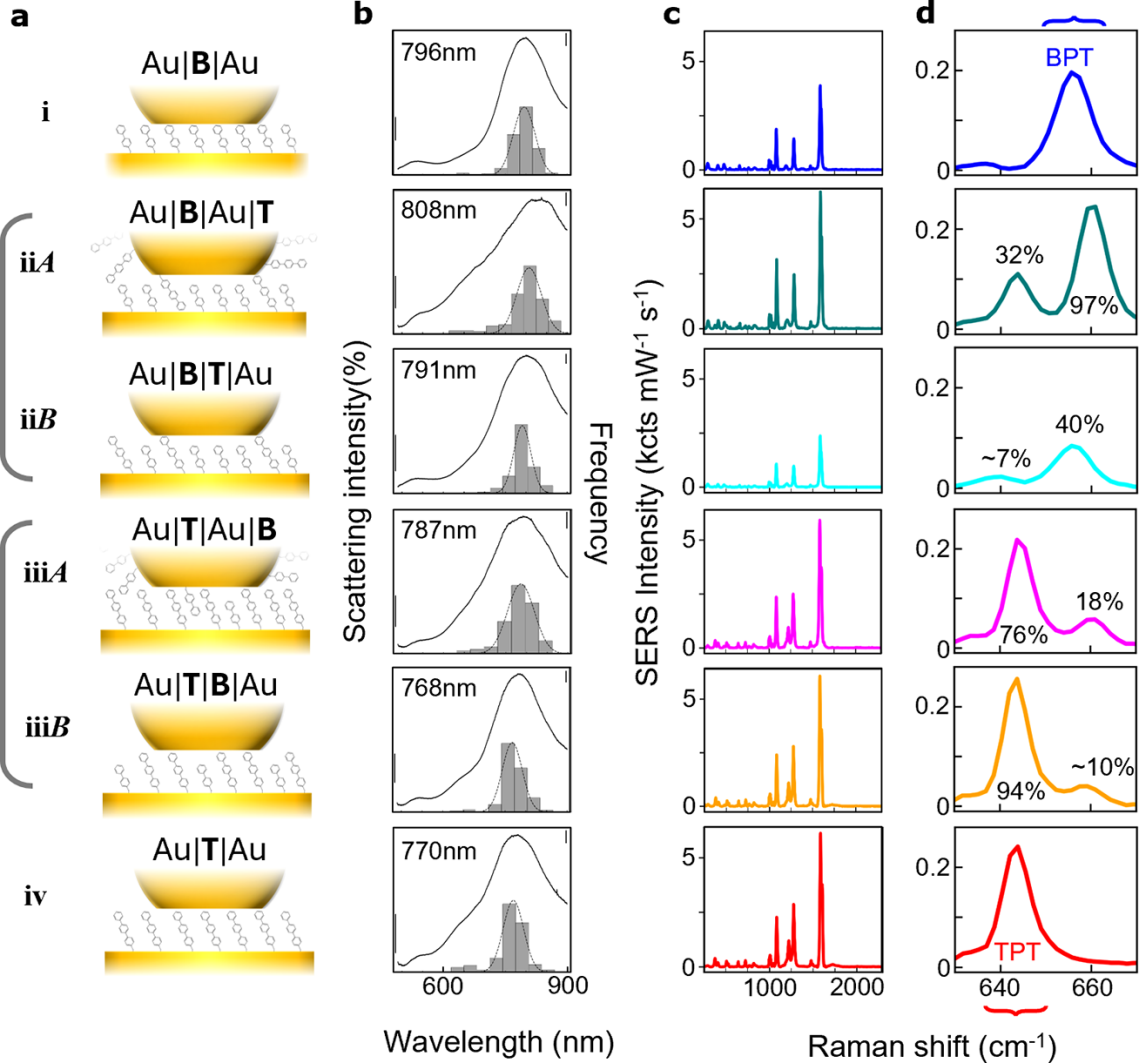
Comparison of molecular accessibility for predeposition (*B*) and postdeposition (*A*). (a) Schematics of (i) Au|B|Au, (ii*A*) Au|B|Au|T, (ii*B*) Au|B|T|Au, (iii*A*) Au|T|Au|B, (iii*B*) Au|T|B|Au, and (iv) Au|T|Au. (b) Histograms of the plasmonic resonant wavelength λ_c_ from dark-field spectra of >200 NPs (right bar = 20) and average spectra from the most frequent bin (left bar = 0.1%). (c, d) Average SERS spectra from >500 NPoMs. Percentages give areas compared to those from the TPT- and BPT-only NPoMs.

We first look at the case where the larger molecule tries to replace the smaller one in the SAM. For postdeposition with Au|B|Au|T (Figure 2ii*A*), the average dark-field (DF) scattering peak of NPoMs unexpectedly redshifts by Δλ_c_ = +12 nm, suggesting an increase in refractive index,^22,23^ while 32% TPT signal appears even though the BPT SAM signal changes little. This clearly demonstrates that TPT molecules diffuse into the gap through the aid of the underside of the AuNP. In addition, the BPT peak is seen to shift to higher wavenumber by 4 cm^–1^ when TPT is added under the NP, suggesting molecule interactions increase at this higher packing. On the other hand, when first mixing the TPT into the BPT SAM, Au|B|T|Au (Figure 2ii*B*), a small blueshift Δλ_c_ = −5 nm from Au|B|Au is seen, while the BPT peak is unshifted.

We also compare with the opposite case where a TPT SAM is replaced with BPT. For Au|T|Au|B, an additional 18% of BPT now enters the nanogap (based on SERS areas) removing little of the TPT SAM (Figure 2iii*A*). At the same time, the average DF peak redshifts by Δλ_c_ ∼ +17 nm relative to Au|T|Au (Figure 2b). In comparison, the Au|T|B|Au (Figure 2iii*B*) introduces <10% BPT and gives negligible redshift from Au|T|Au. Again, it is clear that the presence of the NP still allows the second molecular species to enter the nanogap.

Some caution is needed when directly comparing SERS intensities, as shifts in DF peak position change the optical field confinement and thus SERS enhancement. It is important to note that predeposition (*B*) constrains all molecules to bind to the bottom substrate (as confirmed below), while postdeposition (*A*) gives molecules the option to bind instead to the NP facet. This may account for why more TPT is seen when it is allowed to enter after the NPoM is formed, which is unexpected, given the dense molecular packing of the SAM (as confirmed by STM^24^) that should constrain diffusion into the nanogap. This suggests that the NP surface plays an important role in the molecular diffusion.

We suggest that thiol binding to the Au NP outside the gap (since thiols easily replace surfactant citrate on the Au NPs) is followed by movement of the surface Au atoms that draws each molecule into the gap (as sketched in Figure 2a and further below). We also note that a linear correlation is seen between the plasmon resonance peak position and the ratio of BPT:TPT in the nanogaps (Figure S3), as expected from theoretical models of gap size changes. The greater density of interleaved molecules also increases the refractive index *n*, thus redshifting the resonance (the gap size *d* cannot decrease with additional longer molecules introduced). Theory^23^ shows the DF position scales with $n/\sqrt{d}$ , so that increases in refraction dominate any small change in gap size. If the gap size is assumed to remain constant and we assume the refractive index scales with density, then the molecules become 9% denser in the nanogap, feasible given predicted SAM packings.^24^ Postdeposition thus seems to create chemically asymmetric nanogaps, which remain permeable.

## Binding Location of Molecules within Plasmonic Nanocavities

To resolve where molecules bind within these nanogaps, we employ phenyl-isocyanide (PIC) as a vibrational probe due to its metal-sensitive C≡N stretch, along with our recent capability to glaze single atomic monolayers of different metals onto NPoM facets without reducing their plasmonic enhancement.^25,26^ When coordinated with gold, the Au–C≡N vibration appears at 2180 cm^–1^, while coordination to palladium shifts the mode to 1990 cm^–1^ (Figure 3). By coating the Au substrate with an atomic monolayer of Pd (SI Methods), the orientation of the PIC binding can be quantified (uniquely for this technique), as evident for pure PIC SAMs on either Au or Pd substrates (Figure 3d i,v).

**Figure 3:**
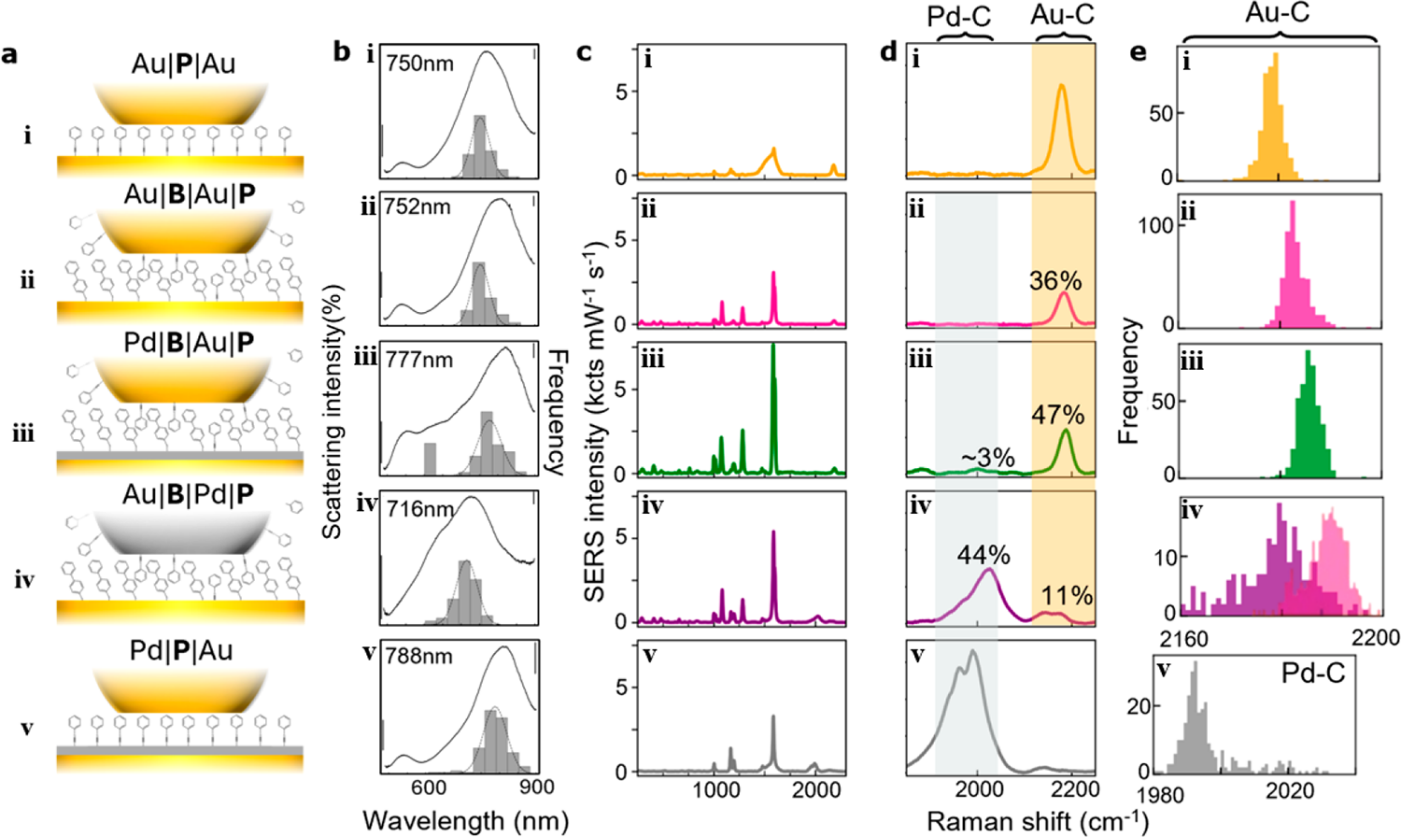
Probing surface-selective coordination of PIC in nanocavities. Data shown for samples of (i) Au|P|Au, (ii) Au|B|Au|P, (iii) Pd|B|Au|P, (iv) Au|B|Pd|P, and (v) Pd|P|Au. (a) Schematics of each. (b) Histograms of the plasmonic resonant wavelength λ_c_ from dark-field spectra of >200 NPs (right bar = 20), and average spectra from the most frequent bin (left bar = 0.1%). (c, d) Average SERS spectra from >500 NPoMs, shaded regions mark Au–PIC (yellow, 2120 cm^–1^) and Pd–PIC (gray, 2000 cm^–1^) binding. Percentages give areas compared to those from the PIC-only NPoMs. (e) Distributions of Raman shifts across single-particle measurements.

When PIC is introduced after the NPoMs are formed with BPT SAMs (Figure 3iii), 47% of Au–C≡N signal emerges but almost nothing attaches to the Pd substrate (<3%). This confirms that molecules entering the gap do so via binding to the NP surface (above the nanogap). This binding asymmetry occurs for both thiol and isocyanide groups. We note that the BPT SAM remains intact (even gaining SERS strength). The entry of PIC (36%) is seen with the Au substrate (Figure 3ii), which is similar to the previous case when replaced with TPT (Figure 2ii*A*). In contrast, when the nanoparticle facet is Pd while the substrate is Au (Figure 3iv), the dominant SERS signal shifts to Pd–C≡N, confirming that PIC binds exclusively to the metal on top of the nanogap after deposition. This then excludes the possibility of nanoparticles moving across the SAM after thiol replacement.

A comparison of the histograms of Au–C≡N Raman shifts from many different NPoMs (Figure 3e) shows they generally exhibit narrow unimodal distributions, indicating consistent molecular binding environments across the ensemble. This implies that PIC consistently localizes to the nanoparticle surface rather than distributing heterogeneously or replacing molecules within the SAM. The only case where replacement is less consistent is using the Pd-coated NP (Figure 3iv), where broader Au–C≡N histograms suggest that SAM morphology is compromised. In this case, a large |Δλ_c_| > 40 nm blueshift is seen, suggesting reduction in gap refractive index (poorer molecular packing) or increased gap size (poorer interpenetration of molecules).

## Dithiol Bridging as a Molecular Diffusion Barrier

The mechanism discussed above depends on single thiol-terminated molecules; hence, we now explore how dithiol molecules bound across the nanogap can modify the replacement process. We thus use biphenyl-4,4-dithiol (BPDT, **D**) SAMs in predeposition, which can bind across the gap and also increase molecular conductivity.^27,28^ We note electrical measurements suggest that <10% of dithiol molecules directly bridge the nanogap.^29^ We then introduce either BPT (**B**) or TPT (**T**) and look in regions of the spectra where the molecules can all be clearly discriminated (Figure 4a,b). Attempting to infiltrate with BPT (Au|D|Au|B) or TPT (Au|D|Au|T) is not effective (fingerprint 620–630 cm^–1^ peak hardly increases, Figure 4a, nor 1605 cm^–1^ Figure 4b); hence, even partial upper thiol binding of the SAM turns off the NP-enhanced replacement mechanism (as depicted in Figure 4c,d).

**Figure 4:**
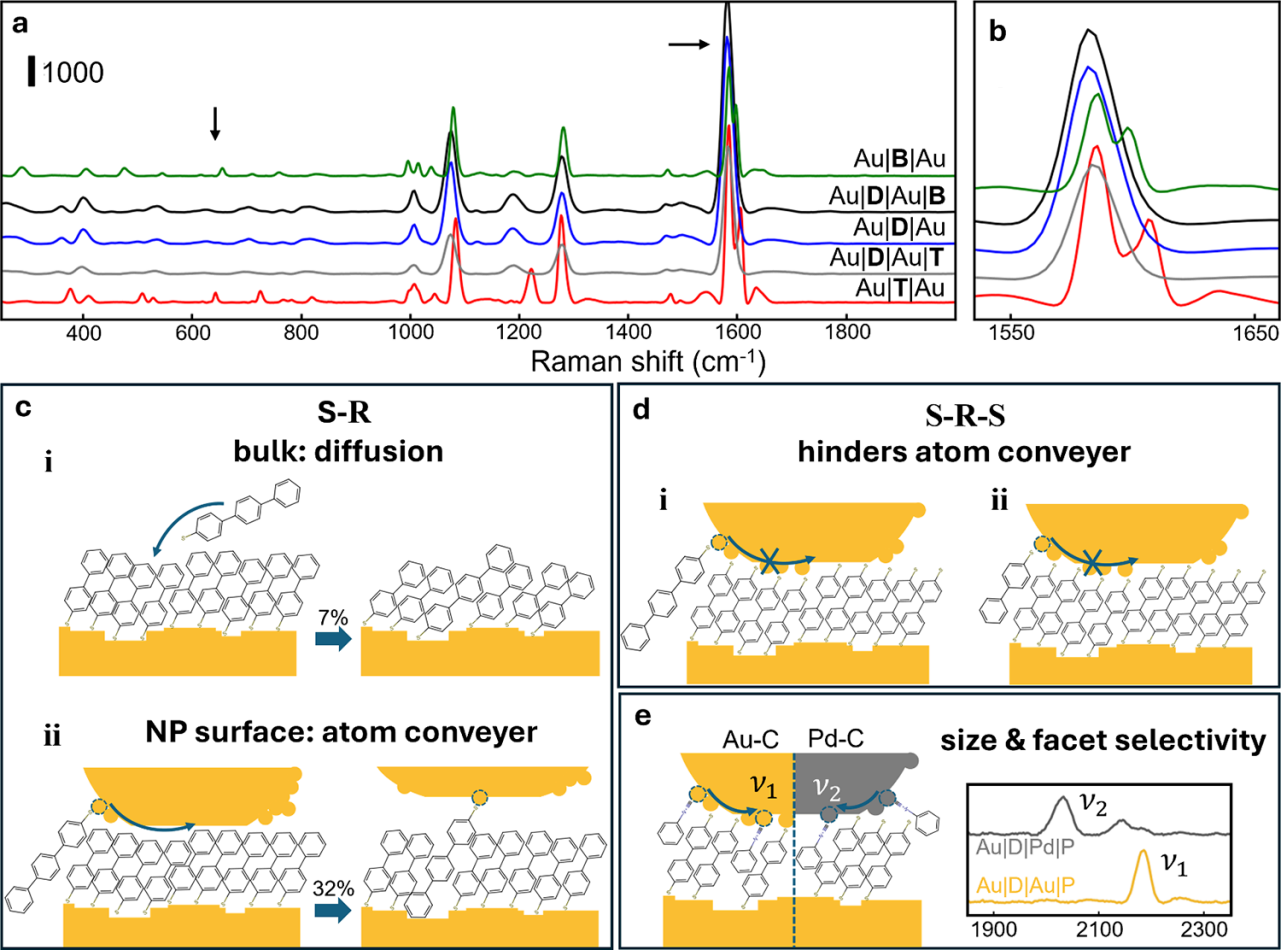
Selective blocking of molecular diffusion within the nanogap. (a,b) Average SERS spectra in key regions for samples as labeled. Arrows indicate BPT/TPT peak. (c) Schematics showing additional atom in-diffusion (conveyer) with NP surface on top of SAM. (d) Schematics illustrating the blocking of the atom conveyer with BPDT SAM. (e) Schematic showing the atom conveyer for both Pd and Au NP surfaces; inset, SERS as labeled.

On the other hand, introducing PIC (**P**) after assembling the BPDT NPoM with an upper surface of either Pd or Au (Figure 4e) shows that such small molecules can still ingress into the gap. As before, the same replacement mechanism operates, since minimal PIC ends up on the lower Au substrate, as seen from the M–C≡N stretch position. The sealing of the nanogap is thus surprisingly selective but shows the additional role of steric constraints. Blocking metal atom movement or reduced molecular flexing may both be involved.

## Discussion of General Mechanism

In general, the replacement of SAM-coated planar open metal surfaces depends mostly on the characteristic cohesion of the molecules and the cost of penetration by a new molecule. Thus, as apparent here, TPT effectively cannot ingress into a BPT SAM (Figure 4c i) on flat Au. However, when a NP sits on top of this SAM, an entirely separate mechanism is introduced, where the movement of the metal atoms drags a metal-bound molecule into the nanogap. Note that, at the 150 μW/μm^2^ intensity levels here, heating is minimal (<30 K). While the environment in these nanogaps can be complex, SERS spectra from thiol SAMs show no evidence of water (OH stretch at 3500 cm^–1^).^30^ An explanation of the NP-assisted diffusion is thus likely to involve the enhanced mobility of the surface metal atoms on the NP (due to their reduced coordination), which can act as a transport conveyer. For instance, instead of single surface Au atoms moving, TEM and STM studies frequently identify chains of atoms moving in concert.^31^ Our data thus suggest that belts of encircling Au atomic chains with molecules attached may rotate around a nanoparticle to bring new molecules into the nanogaps (Figure 4c ii). On the planar surface, such motion is blocked. During the motion, the existing SAM has to temporarily part to the side to provide space for the new molecules to enter. Pressure limitations as the molecular density increases presumably restrict the maximum amount of thiol addition at the nanoparticle surface to <50% (depending on sterics). This provides a picture of much more dynamic motion than commonly supposed at such molecule-metal interfaces on nanoparticles.

The introduction of additional molecules in confined SAMs has a surprisingly weak effect on low wavenumber modes that span the molecules (Figure S1), suggesting they remain only weakly interacting. Alkyl thiol chains may behave differently from the aromatic thiols here;^32^ however, the former possess too low Raman cross sections to be easily tracked in the same manner. Although we see changes in plasmonic resonances here, it is not possible to unambiguously separate the effects of gap size and refractive index (optical permittivity perpendicular to the surface, depending on molecular density and tilt orientation). Additional data when replacing a SAM with the same molecule (Figure S2) is not conclusive but suggests that both the gap size and molecular packing density increase. Shifts or broadening of SERS lines give further information: adding interpenetrating molecules on the top surfaces does not seem to greatly change either (Figure S2e,f). However, the change in PIC C≡N frequency (Figure 3e) between initial SAMs on flat Au and when interpenetrated via the NP suggests extra strain effects.

Additional effects that may be important are clustering of ingress molecules into defects in the SAM (such as molecular crystalline grain boundaries) or around adatom clusters in the upper surface. The lack of strong picocavity^33^ (adatom) effects observed here (because we use laser powers below the threshold for their creation) suggests that these are not prevalent in the diffusion mechanism. Cooperative molecular tilt effects may also play a role, for instance, since BPT apparently binds to gold in a more tilted configuration compared to the slightly more upright TPT.^34,35^ We also note that the large van der Waals attraction of the AuNP to the underlying substrate applies a considerable uniaxial pressure (>100 atm) on the molecular spacers, but no evidence of related compression effects is found here.

In summary, using SERS to quantify the ingress of a second molecule into self-assembled monolayers on a metal, we find dramatically enhanced diffusion when an additional Au nanoparticle is assembled on top. This counterintuitive result can be explained only through the cooperative motion of the surface metal atoms to drag new molecules into the nanogap. We find that this effect does not depend strongly on molecular length, unless a dithiol is included which reduces diffusion by locking the Au NP atoms in place. This result allows new ways to selectively attach molecules to metal surfaces, for instance, allowing a hydrophobic alkanethiol to passivate a planar substrate while selectively introducing active molecules into a nanogap. The ability to exploit this architecture is relevant in a wide range of interactions involving metal-molecule interfaces.

## Supplementary Material



## Data Availability

All data in the current study are available from Cambridge Apollo repository at DOI: 10.17863/CAM.129810.

## Notes

Methods contains all details of experiments and sample fabrication; Figure S1: Comparison of molecular accessibility for pre- and postdeposition; Figure S2: Noncompetitive behavior of molecular exchange; Figure S3: Correlation of DF peak position and gap composition (PDF)
