# Supplementary material for: Surface-Selective Molecular Binding and Replacement Selectivity in Plasmonic Nanocavities
Source: J Phys Chem Lett. 2026 May 7;17(20):5750–6. doi: 10.1021/acs.jpclett.6c00963 (PMC13200228; doi:10.1021/acs.jpclett.6c00963)
Supplement: Supplementary file 1 [file jz6c00963_si_001.pdf]

Supplementary Information

Surface-Selective Molecular Binding and Replacement Selectivity in Plasmonic Nanocavities

Eric S. A. Goerlitzer[1†], Zijia Wu[1†], Aidan Brzakalik[1], Shu Hu[1], Bart de Nijs[2], Jeremy J. Baumberg[1*]

[1] Nanophotonics Centre, Cavendish Laboratory, Dept. of Physics, University of Cambridge, Cambridge, CB3 0US, UK
[2] Physics for Sustainable Chemistry Group, Cavendish Laboratory, Dept. of Physics, University of Cambridge, Cambridge, CB3 0US, UK
* e-mail: jjb12@cam.ac.uk

Supplementary Section: Methods

Supplementary Figures

Fig. S1 | Comparison of molecular accessibility for pre- and post-assembly.. **Error! Bookmark not defined.**

Fig. S2 | Uncompetative behaviour of molecular exchange.. 3

Fig. S3 | Correlation of DF peak position and gap composition.. 3

Supplementary Section: Methods

MATERIALS AND METHODS

Chemicals

Gold (III) chloride trihydrate ($HAuCl_4$, ≥ 99.9%), palladium(II) chloride ($PdCl_2$, 99%), sulfuric acid(H_2SO_4, 95.0-98.0%), citric acid (99.5%), ascorbic acid (AA, ≥ 99%), sodium nitrate ($NaNO_3$), biphenyl-4-thiol(BPT, 97%) tiphenyl-4-thiol (TPT, 97%), and phenyl isocyanide(PIC, 98%), biphenyl-4,4-dithiol (BPDT) and ethanol (99.5%) were purchased from Aldrich-Merck. All chemicals are used without further purification.

SAM sample preparation

SAMs of BPT (denoted **B**), TPT (**T**), and BPDT (**D**) are formed by immersing template-stripped gold substrates, with or without an electrochemically deposited Pd monolayer, in 1 mM ethanolic solutions of the respective thiols for 16 hours, whereas 30 minutes is used for PIC (**P**). Following functionalization, samples are designated in the format substrate|molecule, where, for example, Au|**B** corresponds to an Au substrate modified with BPT. AuNPs or Au@Pd NPs are deposited onto these functionalized substrates via drop-casting. Specifically, 25 µL of nanoparticles (AuNPs or Au@Pd NPs) are deposited onto the samples by drop-casting, and mixed with 1M $NaNO_3$ at a 5:1 volume ratio. The sample are allowed to sit for 20s and rinsed thoroughly with deionized water and dried under a stream of N_2. Secondary SAMs of BPT, TPT, or BPDT are assembled by immersing the pre-functionalized substrates in 1 mM ethanolic thiol solutions for 2 hours; for PIC, a 30-minute immersion is used. The nomenclature used to describe the assembly sequence reflects the order of functionalization and nanoparticle deposition. For example, Au|**B**|**T**|Au denotes a Au substrate initially modified with BPT (16 h), followed by TPT (2 h), and finally decorated with AuNPs; whereas Au|**B**|Au|**T** indicates BPT functionalization, AuNP deposition, and subsequent TPT immersion.

Palladium monolayer deposition

A palladium (Pd) atomic monolayer is electrochemically deposited onto a template-stripped gold (Au) substrate via underpotential deposition (UPD). The process is performed in an aqueous electrolyte containing 0.1 M sulfuric acid (H_2SO_4, 95–98%) and 0.1 mM tetrachloropalladic acid (H_2PdCl_4). A Pt wire functions as the pseudo-reference electrode, and a Au wire is used as the counter electrode. The deposition is carried out by sweeping the potential from 0.3 V to –0.02 V vs the Pt reference electrode at a scan rate of 1 mV/s to enable the reduction of Pd^{2+} ions. A distinct reduction peak appears at 0.05 V vs Pd, preceding the overpotential reduction observed below 0 V, indicating successful monolayer formation.[19,20] The integrated charge under the reduction peak corresponds closely to the theoretical value expected for the deposition of a single Pd atomic monolayer on a (111) surface across the Au substrate. The Pd@Au NPs are prepared by adapting established approaches from the literature. [19,30]

NPoM Scattering and SERS measurements

Scattering and surface-enhanced Raman scattering (SERS) spectra of NPoM structures are collected using a home-built microscope system based on an Olympus BX60 microscope equipped with a 100× dark-field (DF) objective. White-light scattering spectra are acquired using an OceanOptics QEpro spectrometer, while SERS spectra are recorded with an EM-CCD camera (Andor Newton) coupled to a monochromator (Andor Kymera). Samples are first dried and placed on a motorized stage (Prior Scientific) for automated positioning. Illumination through the microscope vertical arm is used both for locating individual NPoMs via scattering images and for acquiring scattering spectra. To correct for chromatic aberrations of the objective, scattering spectra are collected at multiple focal planes. SERS excitation is provided by a single-frequency 633 nm diode laser (Matchbox, Integrated Optics). Calibration of the Raman response is carried out using the Raman signal from silicon wafers. Nanoparticle aggregates, identified by their brighter scattering intensity and distinct morphology, are excluded in real-time using camera imaging. Data processing is conducted in Python using standard libraries. SERS backgrounds were removed using an asymmetric least square (ALS) fitting algorithm, which subtracts a high-degree polynomial from the raw spectra. Additional preprocessing steps ensure the fitted background remains always below the Raman signal.

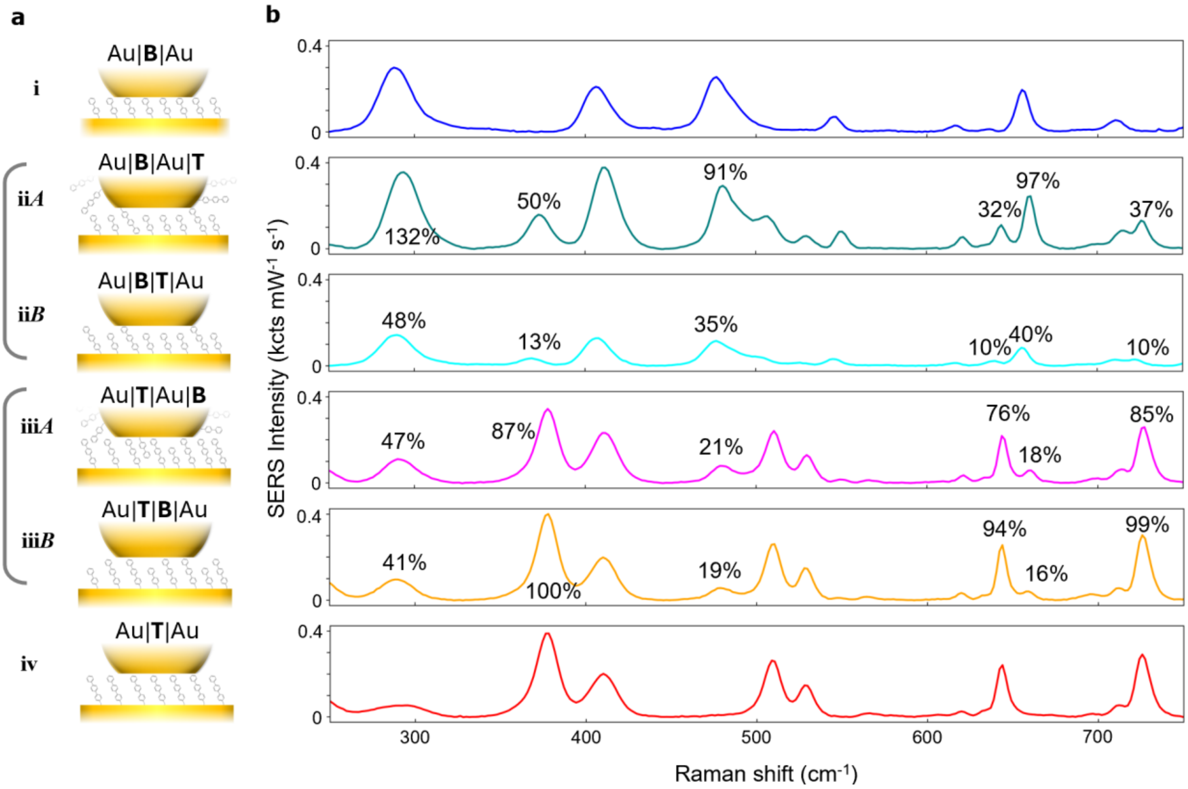

Fig.S1 | Comparison of molecular accessibility for pre- and post-deposition. (a) Schematics of (i) Au|B|Au, (iiA) Au|B|Au|T, (iiB) Au|B|T|Au, (iiiA) Au|T|Au|B, (iiiB) Au|T|B|Au and (iv) Au|T|Au. (b) Average SERS spectra from >500 NPoMs. Percentages give areas compared to those from the TPT- and BPT-only NPoMs.

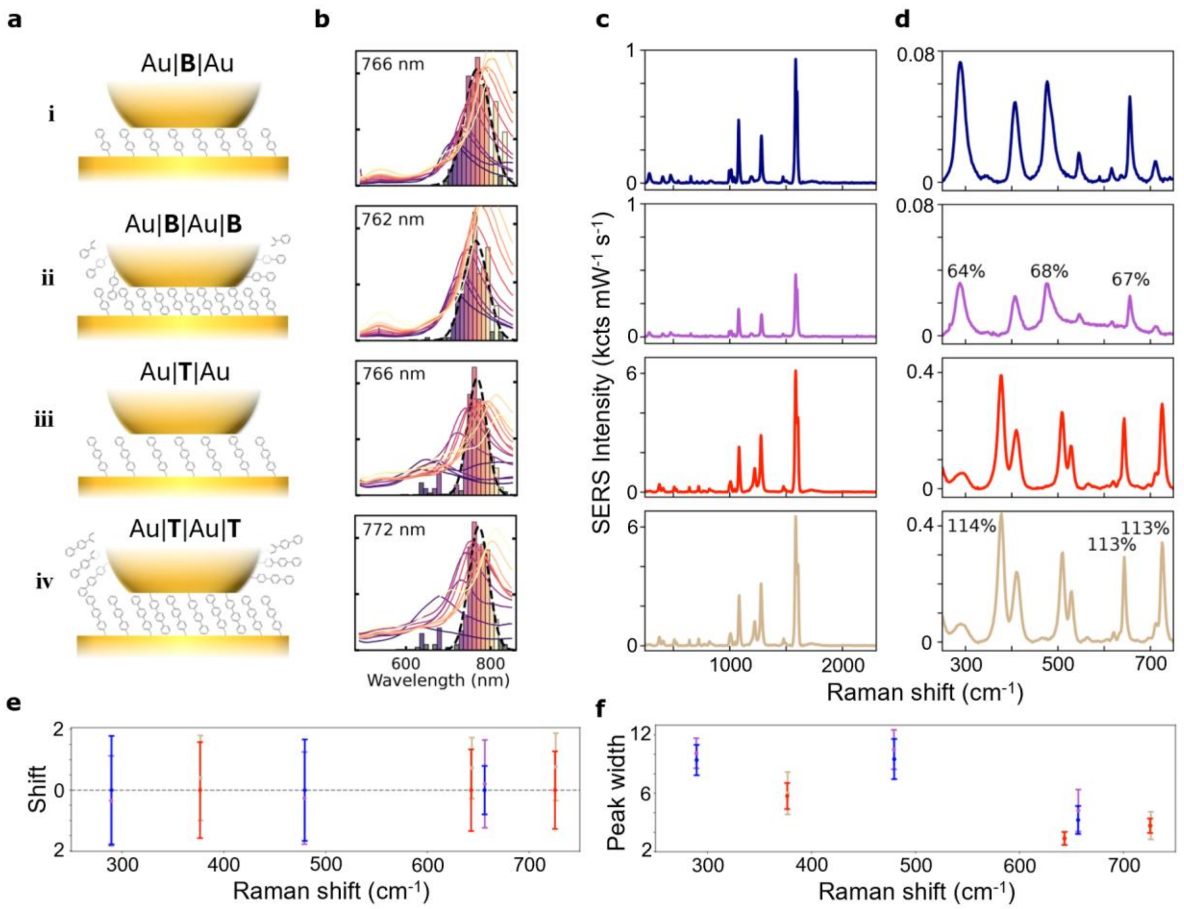

Fig.S2 | Non-competitive molecular exchange in nanogaps. (a) Schematics of (i) Au|B|Au, (ii) Au|B|Au|B, (iii) Au|T|Au and (iv) Au|T|Au|T. (b) Histograms of the plasmonic resonant wavelength λ_c from dark-field spectra of >200 NPs, and average spectra from the most frequent bin. (c,d) Average SERS spectra from >500 NPoMs. Percentages give areas compared to those from the original NPoMs. (e) Shift of each peak in (d) after pre-deposition, error bars give standard deviation between individual NPoMs. f) SERS peak width in (d,e), error bars give the standard deviation between individual NPoMs.

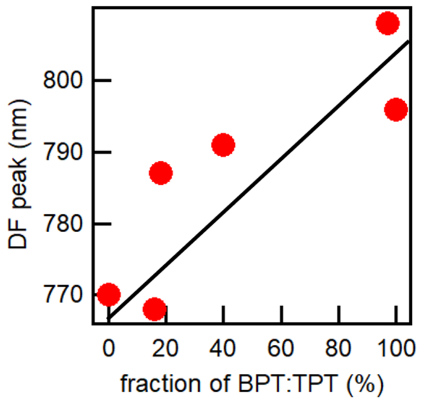

Fig.S3 | Correlation of DF peak position and gap composition. Extracted dark-field peak position *vs* the ratio of BPT to total BPT+TPT extracted from SERS signal of many NPoMs, using data from Fig.2. Line shows result from simulations of NPoM (see [11]) using 80nm AuNPs, with 20nm facet and thickness from tilted BPT and TPT SAMs.
